# *Terminalia bellirica* (Gaertn.) Roxb. Extract and Gallic Acid Attenuate LPS-Induced Inflammation and Oxidative Stress via MAPK/NF-*κ*B and Akt/AMPK/Nrf2 Pathways

**DOI:** 10.1155/2018/9364364

**Published:** 2018-11-08

**Authors:** Miori Tanaka, Yoshimi Kishimoto, Mizuho Sasaki, Akari Sato, Tomoyasu Kamiya, Kazuo Kondo, Kaoruko Iida

**Affiliations:** ^1^Department of Food and Nutritional Sciences, Graduate School of Humanities and Sciences, Ochanomizu University, 2-1-1 Otsuka, Bunkyo-ku, Tokyo 112-8610, Japan; ^2^Endowed Research Department “Food for Health”, Ochanomizu University, 2-1-1 Otsuka, Bunkyo-ku, Tokyo 112-8610, Japan; ^3^Research and Development Division, Toyo Shinyaku Co Ltd, 7-28 Yayoigaoka, Tosu-shi, Saga 841-0005, Japan; ^4^Institute of Life Innovation Studies, Toyo University, 1-1-1 Izumino, Itakura-machi, Ora-gun, Gunma 374-0193, Japan; ^5^Institute for Human Life Innovation, Ochanomizu University, 2-1-1 Otsuka, Bunkyo-ku, Tokyo 112-8610, Japan

## Abstract

Excessive oxidative stress plays a critical role in the progression of various diseases. Recently, we showed that *Terminalia bellirica* (Gaertn.) Roxb. extract (TBE) inhibits inflammatory response and reactive oxygen species (ROS) production in THP-1 macrophages. However, molecular mechanisms underlying anti-inflammatory and antioxidant activities of TBE and its major polyphenolic compounds gallic acid (GA) and ellagic acid (EA) remain unclear. We found that TBE and GA attenuated LPS-induced inflammatory mediator expression, ROS production, and activation of mitogen-activated protein kinase (MAPK) and nuclear factor-kappa B (NF-*κ*B) in RAW 264 macrophages. Furthermore, TBE and GA increased antioxidant enzyme expression along with upstream mediators nuclear factor erythroid-2-related factor 2 (Nrf2), Akt, and AMP-activated protein kinase (AMPK). Importantly, knockdown of Nrf2 by siRNA and specific inhibition of Akt and AMPK significantly reduced antioxidant enzyme expression induced by TBE and GA. Finally, *in vivo* effects on histopathology and gene expression were assessed in tissues collected after intraperitoneal injection of LPS with or without TBE treatment. TBE enhanced antioxidant enzyme expression and improved acute kidney injury in LPS-shock model mice. In conclusion, TBE and GA exert protective effects against inflammation and oxidative stress by suppressing MAPK/NF-*κ*B pathway and by activating Akt/AMPK/Nrf2 pathway. These results suggest that TBE and GA might be effective for the treatment of inflammation-related diseases.

## 1. Introduction

Inflammation is an innate defense system of the human body against environmental injuries and pathogens. However, excessive inflammation contributes to serious tissue damage and the development of human diseases such as atherosclerosis, diabetes, and cancer [1]. Many investigations have suggested a relationship between activation of macrophages, which are pivotal immune cells for regulating inflammation, and human inflammatory disorders [2]. Macrophage-mediated inflammatory responses are typically triggered by pathogen-associated molecular patterns (PAMPs) recognized by toll-like receptors (TLRs) [3, 4]. Lipopolysaccharide (LPS), the most representative PAMPs produced by gram-negative bacteria, is a ligand of TLR4 that can activate downstream signaling pathways such as mitogen-activated protein kinase (MAPK) and nuclear factor-kappa B (NF-*κ*B), eventually leading to the generation of proinflammatory cytokines, chemokines, nitric oxide (NO), and reactive oxygen species (ROS) [5, 6]. ROS have been shown to activate MAPK and NF-*κ*B pathways and result in inflammation [7, 8]. Indeed, inhibiting ROS production was reported to suppress inflammatory mediator expression in macrophages [9]. Therefore, the modulation of cellular redox status is a key regulator of inflammatory response and important for providing a therapeutic strategy against inflammation-related diseases.

Oxidative stress results from an imbalance between excessive amounts of ROS and the antioxidant defense system. The human body has the ability to protect cells against oxidative damage with endogenous antioxidant enzymes (e.g., heme oxygenase-1 (HO-1), catalase, NADPH quinone oxidoreductase 1 (NQO1), and glutamate-cysteine ligase modifier subunit (GCLM)). In addition, exogenous antioxidants such as polyphenol, and vitamins C and E are obtained through diet. The consumption of some polyphenols has been reported to induce antioxidant enzyme expression and activity [10, 11]. Nuclear factor erythroid-2-related factor 2 (Nrf2) is a primary transcription factor that regulates antioxidant enzyme expression. Nrf2 plays an imperative role in cellular defense against oxidative stress and inflammation by activating antioxidant cascades [12–14]. The transcriptional activity of Nrf2 protein is suppressed by its negative regulator Kelch-like ECH-associated protein 1 (Keap1) under homeostatic conditions; however, upon oxidative stress, Nrf2 translocates to the nucleus where it binds to the antioxidant responsive element (ARE) [15]. In addition to the Keap1/Nrf2 pathway, phosphorylation of Nrf2 by several cytosolic kinases (e.g., protein kinase C, phosphoinositide 3-kinase (PI3K)/Akt, and MAPK) has been shown to facilitate release of Nrf2 from Keap1 and subsequent antioxidant signal cascades [16].

PI3K and its downstream mediator Akt constitute a critical signaling pathway regulating a variety of biological processes such as cell growth, proliferation, apoptosis, and protein synthesis [17]. PI3K/Akt pathway has also been implicated in Nrf2-mediated antioxidant response [16]. AMP-activated protein kinase (AMPK), a heterotrimeric serine/threonine kinase, is a crucial energy sensor of cellular metabolism in response to various metabolic stresses such as oxidative stress, inflammation, and hypoxia [18]. Previous studies suggested that AMPK can stimulate nuclear accumulation of Nrf2 [19] and protect against inflammation by inhibiting NF-*κ*B signaling pathway [20]. Moreover, Sag et al. [21] demonstrated that AMPK deletion dramatically increased inflammatory mediator expression in LPS-stimulated macrophages. Several polyphenols (e.g., resveratrol, quercetin, and catechins) have also been shown to downregulate NF-*κ*B signaling and inflammatory response through the activation of PI3K/Akt and AMPK [22–24]. Although mechanistic connections between both PI3K/Akt and AMPK pathways and inflammation have been frequently reported, potential roles of PI3K/Akt and AMPK in antioxidant effects induced by dietary-derived polyphenols mostly remain unclear.

*Terminalia bellirica* (Gaertn.) Roxb. extract (TBE) is obtained from the fruit of *T. bellirica* tree, which is distributed throughout Southeast Asia and used as a folk medicine for diabetes, rheumatism, and hypertension in traditional Indian Ayurvedic medicine [25]. Multiple studies have suggested antiobesity, hypoglycemic [26], hypolipidemic [27], and antihypertensive [28] properties of the fruit. The major polyphenolic compounds of this fruit are reported to be gallic acid (GA), ellagic acid (EA), and gallate esters [29]. GA has been shown to exert curative effects against obesity-related atherosclerosis and insulin resistance via the activation of AMPK [30, 31]. Our previous report revealed that TBE inhibited inflammatory mediator expression and ROS production in THP-1 macrophages [32], but there is little information about anti-inflammatory and antioxidant activities of TBE and underlying mechanisms in this process. This study examined protective effects of TBE and its major bioactive ingredients on inflammation and oxidative stress, as well as the underlying molecular mechanisms, by utilizing LPS-stimulated macrophages and LPS-shock model mice.

## 2. Materials and Methods

### 2.1. Reagents

TBE was provided by Toyo Shinyaku Co. Ltd. (Saga, Japan). The total polyphenol content of TBE powder was 23.1% in our previous study [32]. The powder was dissolved in deionized water at 40 mg/mL and used in experiments. GA, EA, LPS (from *Escherichia coli* O11:B4), palmitic acid, Hank's balanced salt solution (HBSS), 3-(4,5-dimethylthiazol-2-y1)-2,5-diphenyltetrazoliumbromide (MTT), L-Arginine, LY294002, and compound C were purchased from Sigma-Aldrich (St Louis, MO, USA). Dulbecco's modified eagle medium (DMEM), fetal bovine serum (FBS), and penicillin/streptomycin were obtained from Gibco (Life Technologies, Carlsbad, CA, USA). Diaminofluorescein-2 (DAF-2) was acquired from Sekisui Medical (Tokyo, Japan). 5-(And-6)-chloromethyl-2′,7′-dichlorohydrofluorescein diacetate (CM-H_2_DCFDA), Nrf2 Stealth RNAi siRNA, and Lipofectamine RNAiMAX were purchased from Thermo Fisher Scientific (Waltham, MA, USA).

### 2.2. HPLC Analysis of Phenolic Compounds

HPLC analysis of phenolic components in TBE was performed. The TBE stock solution was diluted with 50% ethanol (*v*/*v*) at 1 mg/mL, filtered through a 0.45 *μ*m PTFE filter, and injected into a UK-C18 HT (3 *μ*m particle size, 3 × 100 mm, Imtakt Corporation) HPLC column. The mobile phases were 1% formic acid in water (*v*/*v*) as eluent A, and 99% acetonitrile, 1% formic acid (*v*/*v*) as eluent B. The gradient program was as follows: 0–3 min, 95% A, 5% B; 3–11.5 min, 75% A, 25% B; 11.5–13.5 min, 20% A, 80% B; and 13.5–17 min, 95% A, 5% B. The detection was UV absorbance at 276 nm. GA and EA were quantified by comparison with a multipoint calibration curve obtained from the corresponding standard (GA (Sigma-Aldrich) and EA (Wako Pure Chemical)).

### 2.3. Cell Culture and Treatment

The murine macrophage cell line RAW 264 was obtained from the RIKEN Cell Bank (Ibaraki, Japan). Cells were cultured in DMEM supplemented with 10% FBS, 100 U/mL penicillin, and 100 *μ*g/mL streptomycin at 37°C and 5% CO_2_. For fatty acid treatment, palmitic acid was dissolved in 100 mM NaOH for 15 min at 70°C. 100 mM palmitic acid solution was then mixed with prewarmed fatty acid-free BSA (10% in DMEM) to yield 8 mM palmitic acid stock solution. The solution was incubated for 15 min at 55°C and stored at −20°C until use.

### 2.4. Cell Viability

Cell viability was determined by MTT assay. RAW 264 macrophages were seeded in 24-well plates at a density of 3.5 × 10^5^ cells/mL and incubated for 48 h at 37°C and 5% CO_2_. Cells were treated with 100–400 *μ*g/mL TBE or 11.5–46 μg/mL GA (same concentrations as the amount contained in TBE according to the HPLC analysis) for 8 h. Afterwards, fresh media containing MTT (0.5 mg/mL) was added to each well and incubated for 3 h. The culture medium was carefully removed and the resulting formazan crystals were dissolved in DMSO (250 *μ*L/well). The absorbance was measured at 540 nm using a microplate reader (BioTek Instruments, Tokyo, Japan).

### 2.5. Real-Time RT-PCR

RAW 264 macrophages were seeded in 12-well plates at a density of 3.5 × 10^5^ cells/mL and incubated for 48 h at 37°C and 5% CO_2_. Cells were pretreated with 100–400 *μ*g/mL TBE, 46 *μ*g/mL GA, or 1.6 *μ*g/mL EA (same concentration as the amount contained in TBE according to the HPLC analysis) for 1 h and then LPS (100 ng/mL) was added and incubation was continued for an additional 4 h. For palmitic acid treatment, cells were pretreated with 400 *μ*g/mL TBE or 46 *μ*g/mL GA for 1 h and then palmitic acid (400 *μ*M) was added and incubated for 12 h. Total cellular RNA was extracted using RNAiso Plus (Takara Bio, Shiga, Japan) according to the manufacturer's instructions. We reverse transcribed first-stand complementary DNA from 2 *μ*g of total RNA using a High Capacity cDNA Reverse Transcription Kit (Applied Biosystems, Foster City, CA, USA). Real-time PCR was performed on a StepOnePlus Real-Time PCR System (Applied Biosystems) using Power SYBR Green PCR mix (Applied Biosystems). The results are expressed as the copy number ratio of the target mRNA to GAPDH mRNA. Primers of genes encoding tumor necrosis factor-alpha (TNF-*α*) (*Tnf*), interleukin-1 beta (IL-1*β*) (*Il1b*), IL-6 (*Il6*), inducible nitric oxide synthase (iNOS) (*Nos2*), monocyte chemoattractant protein-1 (MCP-1) (*Ccl2*), class A scavenger receptor (SR-A) (*Msr1*), HO-1 (*Hmox1*), catalase (*Cat*), NQO1 (*Nqo1*), GCLM (*Gclm*), and Nrf2 (*Nfe2l2*) were obtained from Sigma-Aldrich. Primer sequences are listed in Supplementary Table S1.

### 2.6. Western Blot Analysis

RAW 264 macrophages were seeded in 6-well plates at a density of 3.5 × 10^5^ cells/mL and incubated for 72 h at 37°C and 5% CO_2_. Cells were pretreated with 100–400 *μ*g/mL TBE for 1 h and then LPS (100 ng/mL) was added and incubation was continued for 0.5, 2, and 6 h. Total protein was extracted using M-PER Mammalian Protein Extraction Reagent (Thermo Fisher Scientific). Cytosolic and nuclear fractionation was performed using NE-PER Nuclear and Cytoplasmic Extraction Reagents (Thermo Fisher Scientific). Equal amounts of cellular proteins were electrophoresed on 10% sodium dodecyl sulfate-polyacrylamide gels and transferred to Immobilon-P membranes (Merck Millipore, Billerica, MA, USA). Membranes were blocked with 5% skim milk or 5% BSA and incubated with primary antibodies against iNOS (Sigma-Aldrich), SR-A, NF-*κ*B p65, I*κ*B-*α*, Nrf2, Lamin B (Santa Cruz Biotechnology, Dallas, TX, USA), HO-1 (Enzo Life Sciences, Farmingdale, NY, USA), catalase (AbFrontier, Seoul, Korea), p38 MAPK, c-jun N-terminal kinase (JNK), extracellular signal-regulated kinase (ERK), Akt, AMPK, *β*-actin, and GAPDH (Cell Signaling Technology, Danvers, MA, USA). After washing with TBS-T, membranes were incubated with peroxidase-conjugated secondary antibodies: anti-rabbit (Cell Signaling Technology), anti-mouse, and anti-goat (Santa Cruz Biotechnology). Chemiluminescent detection of specific proteins was developed with ECL Select Western Blotting Detection Reagent (GE Healthcare, Little Chalfont, UK). All signals were detected by an ImageQuant LAS 4000 system (Fujifilm, Tokyo, Japan).

### 2.7. Measurement of NO Production

NO levels were measured using DAF-2, a sensitive fluorescent dye for the detection of NO. RAW 264 macrophages were seeded in 24-well plates at a density of 3.5 × 10^5^ cells/mL and incubated for 48 h at 37°C and 5% CO_2_. Cells were pretreated with 100–400 *μ*g/mL TBE for 1 h and then LPS (100 ng/mL) was added and incubation was continued for 6 h. Next, 10 *μ*M DAF-2 and 5 mM L-arginine in fresh HBSS was added and cells were incubated for an additional 2 h at 37°C. After incubation, the fluorescent intensity of culture supernatant was determined at 495 nm excitation and 515 nm emission using a microplate reader.

### 2.8. Measurement of Intracellular ROS Production

Intracellular ROS levels were determined by measuring the oxidative conversion of cell permeable 5-(and 6)-chloromethyl-2′,7′-dichlorohydrofluorescein diacetate (CM-H_2_DCFDA) to dichlorofluorescein (DCF), a fluorescent product. RAW 264 macrophages were seeded in 24-well plates at a density of 3.5 × 10^5^ cells/mL and incubated for 48 h at 37°C and 5% CO_2_. Cells were pretreated with 100–400 *μ*g/mL TBE or 11.5–46 *μ*g/mL GA for 1 h and then LPS (100 ng/mL) was added and incubation was continued for 7 h. After washing cells with HBSS, 10 *μ*M CM-H_2_DCFDA/HBSS was added and incubated for further 30 min at 37°C. The fluorescent intensity was detected at 492 nm excitation and 517 nm emission with a microplate reader. DCF fluorescence images were acquired under a BZ-X710 fluorescence microscope (20x objective lens, KEYENCE, Osaka, Japan).

### 2.9. Small Interfering RNA Transfection

RAW 264 macrophages were transfected with 60 nM Nrf2 siRNA or negative control (NC) siRNA using Lipofectamine RNAiMAX for 48 h. After transfection, cells were pretreated with 400 *μ*g/mL TBE or 46 *μ*g/mL GA for 1 h and then LPS (100 ng/mL) was added and incubation was continued for 4 h. Real-time RT-PCR was performed as described above.

### 2.10. Animal Experiments

Male ICR mice (8 weeks, 36–43 g) were purchased from Sankyo Labo Service Corporation (Tokyo, Japan). All animal experiments were approved by the Animal Ethics Committee of Ochanomizu University (approved number; 17025R) and performed in accordance with Act on Welfare and Management of Animals. Mice were randomly divided into two groups: LPS (*n* = 6) and LPS + TBE (*n* = 6). TBE (400 mg/kg body weight, dissolved in water) was orally administrated to mice once a day for 3 consecutive days. One hour after the last administration, all mice were intraperitoneally injected with LPS (2 mg/kg body weight). Kidney tissues were collected at 24 h post LPS injection and subsequently used for real-time RT-PCR and histopathological examination.

### 2.11. Histopathological Examination

Kidney tissues from mice were fixed with 3.7% formaldehyde, embedded in paraffin, and cut into 5 *μ*m sections. Sections were stained with hematoxylin and eosin (HE) and viewed under a BZ-X710 fluorescence microscope (40x objective lens). The extent of interstitial hyperemia was scored using a semiquantitative scale designed to evaluate interstitial hyperemia. The score (ranging between 1 and 4) was judged as follows: 1, normal kidney; 2, mild hyperemia; 3, moderate hyperemia; and 4, severe hyperemia by two blinded investigators. The percentage of glomerular capillary narrowing was also analyzed by two investigators.

### 2.12. Statistical Analysis

Comparisons between treatment groups were performed using an unpaired *t*-test or one-way analysis of variance (ANOVA) followed by Tukey's post hoc test. Differences were considered statistically significant when *p* < 0.05. Statistical analyses were performed using the GraphPad Prism 5 software package (GraphPad Software, La Jolla, CA, USA).

## 3. Results

### 3.1. Polyphenol Composition of TBE by HPLC Analysis

**Figure 1:**
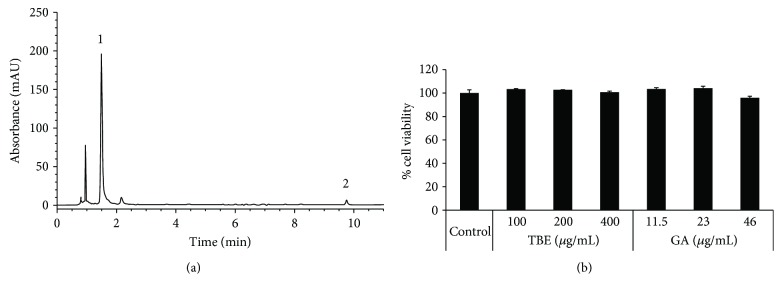
HPLC-ESI/MS chromatogram of TBE solution and effects of TBE and GA on cell viability in macrophages. (a) Peaks indicate (1) gallic acid and (2) ellagic acid. (b) RAW 264 cells were treated with 100–400 *μ*g/mL TBE or 11.5–46 *μ*g/mL GA for 8 h, and then cell viability was determined by MTT assay. Data represent mean ± SD, *n* = 3.

To determine the polyphenol composition of TBE, we performed HPLC analysis. According to the HPLC analytical plot, contents of GA and EA in TBE solution (40 mg/mL) were 4.6 mg/mL and 0.16 mg/mL, respectively (Figure 1(a)). Thus, the contents of GA and EA in TBE powder were calculated to be 115 mg/g and 4 mg/g, suggesting that gallic acid is the major polyphenolic compound of TBE.

### 3.2. Effects of TBE and GA on Cell Viability in RAW 264 Cells

We first analyzed the effects of TBE and GA on the viability of RAW 264 macrophages by MTT assay. As shown in Figure 1(b), no cytotoxic effect was observed when cells were exposed to TBE (100–400 *μ*g/mL) or GA (11.5–46 *μ*g/mL) for 8 h.

### 3.3. TBE Exerted Anti-Inflammatory Effect in LPS-Stimulated Macrophages

**Figure 2:**
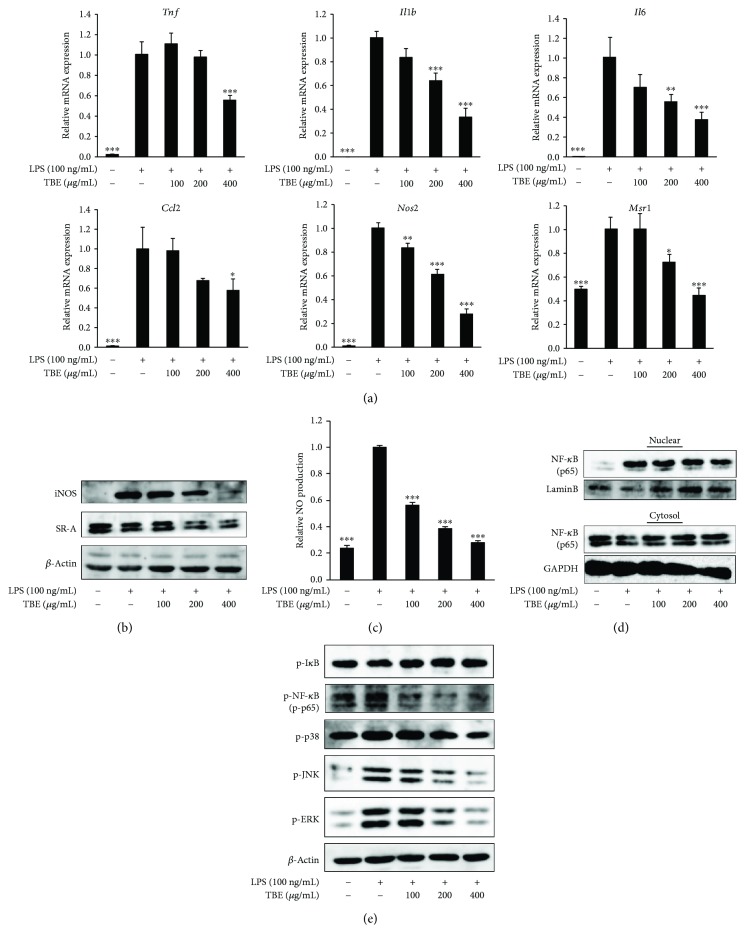
Effect of TBE on inflammatory mediator expression and inflammatory signaling pathway in LPS-stimulated macrophages. (a) RAW 264 cells were pretreated with 100–400 *μ*g/mL TBE for 1 h, followed by treatment with 100 ng/mL LPS for 4 h. The mRNA levels of *Tnf*, *Il1b*, *Il6*, *Ccl2*, *Nos2*, and *Msr1* were detected by real-time RT-PCR. (b, c) RAW 264 cells were pretreated with 100–400 *μ*g/mL TBE for 1 h, followed by treatment with 100 ng/mL LPS for 6 h. (b) The protein levels of iNOS and SR-A were detected by Western blotting. (c) NO levels in the culture medium were measured using DAF-2. (d) RAW 264 cells were pretreated with 100–400 *μ*g/mL TBE for 1 h, followed by treatment with 100 ng/mL LPS for 2 h. NF-*κ*B activation was determined by measuring cytosolic and nuclear p65 levels. (e) RAW 264 cells were pretreated with 100–400 *μ*g/mL TBE for 1 h, followed by treatment with 100 ng/mL LPS for 0.5 h. NF-*κ*B and MAPK activation were assessed by measuring p-I*κ*B, P-NF-*κ*B, p-p38, p-JNK, and p-ERK. Data represent mean ± SD, *n* = 3 (^∗^ *p* < 0.05, ^∗∗^ *p* < 0.01, ^∗∗∗^ *p* < 0.001 compared to LPS group).

As the production of inflammatory mediators is a well-known response to LPS stimulation in macrophages, we examined the effect of TBE on inflammatory mediator expression. TBE significantly reduced LPS-induced mRNA expression of TNF-*α*, IL-1*β*, IL-6, MCP-1, iNOS, and SR-A, as well as protein expression of iNOS and SR-A (Figures 2(a) and 2(b)). NO production was also examined because excessive NO production is associated with inflammatory responses. As shown in Figure 2(c), LPS caused a considerable release of NO, but TBE significantly decreased the level of NO production. To evaluate regulatory mechanisms of TBE on inflammatory signaling pathways, we analyzed its inhibitory effect on NF-*κ*B and MAPK activation. Western blot analysis revealed that the nuclear translocation of NF-*κ*B p65 and phosphorylation of NF-*κ*B p65, p38, JNK, and ERK were increased by LPS. Treatment with TBE effectively inhibited the nuclear translocation of NF-*κ*B and phosphorylation of all these proteins, whereas TBE did not affect phosphorylation of I*κ*B (Figures 2(d) and 2(e)).

### 3.4. Effects of GA and EA on Inflammatory Mediator Expression in LPS- and Palmitic Acid-Stimulated Macrophages

**Figure 3:**
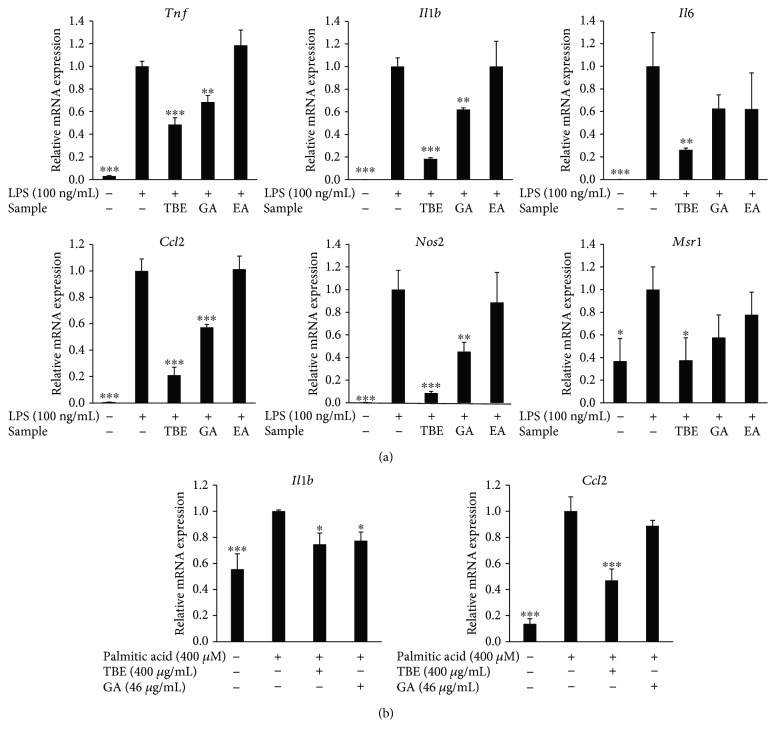
Effects of TBE, GA, and EA on inflammatory mediator expression in LPS- and palmitic acid-stimulated macrophages. (a) RAW 264 cells were pretreated with 400 *μ*g/mL TBE, 46 *μ*g/mL GA, or 1.6 *μ*g/mL EA for 1 h, followed by treatment with 100 ng/mL LPS for 4 h. The mRNA levels of *Tnf*, *Il1b*, *Il6*, *Ccl2*, *Nos2*, and *Msr1* were detected by real-time RT-PCR. (b) RAW 264 cells were pretreated with 400 *μ*g/mL TBE or 46 *μ*g/mL GA for 1 h, followed by treatment with 400 *μ*M palmitic acid for 12 h. The mRNA levels of *Il1b* and *Ccl2* were detected by real-time RT-PCR. Data represent mean ± SD, *n* = 3 (^∗^ *p* < 0.05, ^∗∗^ *p* < 0.01, ^∗∗∗^ *p* < 0.001 compared to LPS or palmitic acid group).

The present HPLC analysis showed that TBE contains GA and EA and that GA is the major polyphenolic compound of TBE. Therefore, we assessed the effects of GA and EA in TBE on inflammatory mediator expression. As shown in Figure 3(a), LPS upregulated the expression of TNF-*α*, IL-1*β*, IL-6, MCP-1, iNOS, and SR-A in macrophages. GA treatment significantly reduced LPS-induced expression of TNF-*α*, IL-1*β*, MCP-1, and iNOS as well as TBE, but EA had no effect. Similar to LPS, saturated fatty acid such as palmitic acid is known to exert proinflammatory activity in macrophages via TLR4. As shown in Figure 3(b), palmitic acid increased the mRNA expression of IL-1*β* and MCP-1, while TBE significantly suppressed the expression of these genes and GA reduced IL-1*β* expression.

### 3.5. TBE and GA Suppressed ROS Production in LPS-Stimulated Macrophages

**Figure 4:**
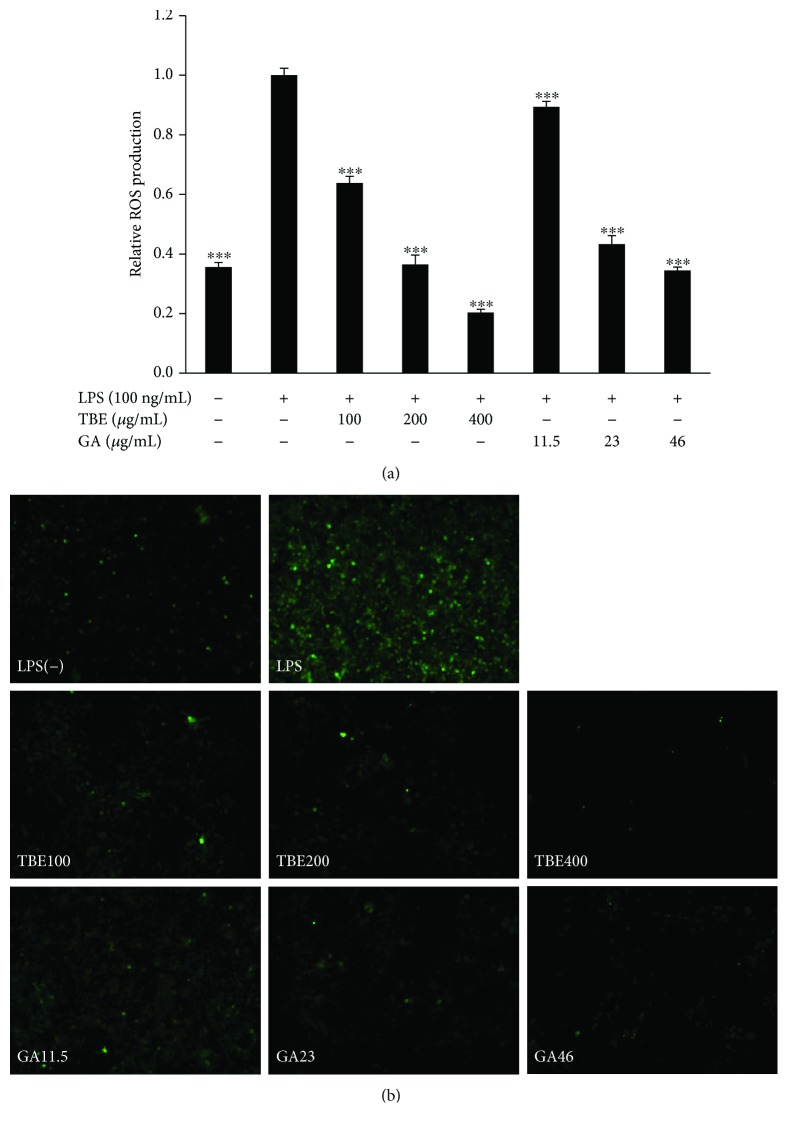
Effects of TBE and GA on ROS production in LPS-stimulated macrophages. (a, b) RAW 264 cells were pretreated with 100–400 *μ*g/mL TBE or 11.5–46 *μ*g/mL GA for 1 h, followed by treatment with 100 ng/mL LPS for 7 h. Intracellular ROS levels were measured using CM-H_2_DCFDA. Data represent mean ± SD, *n* = 3 (^∗∗∗^ *p* < 0.001 compared to LPS group).

Elimination of ROS production is important for controlling inflammatory response. To confirm the antioxidant effects of TBE and GA in LPS-stimulated macrophages, we measured intracellular ROS production using CM-H_2_DCFDA. As shown in Figures 4(a) and 4(b), ROS production was greatly increased by LPS, showing obvious green fluorescence, while TBE and GA significantly suppressed the level of ROS production in a dose-dependent manner.

### 3.6. TBE Enhanced the Antioxidant Defense System in LPS-Stimulated Macrophages

**Figure 5:**
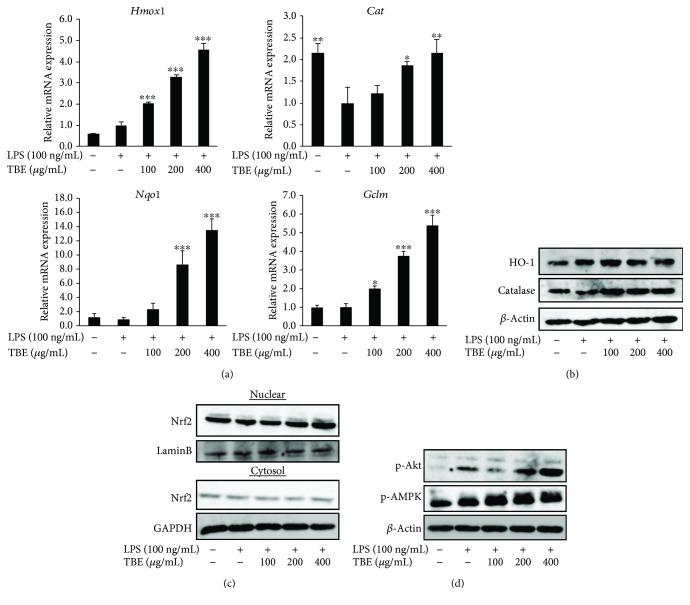
Effect of TBE on antioxidant enzyme expression and antioxidant signaling pathway in LPS-stimulated macrophages. (a) RAW 264 cells were pretreated with 100–400 *μ*g/mL TBE for 1 h, followed by treatment with 100 ng/mL LPS for 4 h. The mRNA levels of *Hmox1*, *Cat*, *Nqo1*, and *Gclm* were detected by real-time RT-PCR. (b) RAW 264 cells were pretreated with 100–400 *μ*g/mL TBE for 1 h, followed by treatment with 100 ng/mL LPS for 6 h. The protein levels of HO-1 and catalase were detected by Western blotting. (c) RAW 264 cells were pretreated with 100–400 *μ*g/mL TBE for 1 h, followed by treatment with 100 ng/mL LPS for 2 h. Nrf2 activation was determined by measuring cytosolic and nuclear Nrf2 levels. (d) RAW 264 cells were pretreated with 100–400 *μ*g/mL TBE for 1 h, followed by treatment with 100 ng/mL LPS for 0.5 h. Akt and AMPK activation was detected by Western blotting. Data represent mean ± SD, *n* = 3 (^∗^ *p* < 0.05, ^∗∗^ *p* < 0.01, ^∗∗∗^ *p* < 0.001 compared to LPS group).

The antioxidant defense system, such as antioxidant enzymes, is important for the suppression of oxidative stress and inflammatory response. We found that LPS had almost no effect on the mRNA or protein expression of HO-1, NQO1, and GCLM and decreased the expression of catalase. Treatment with TBE significantly upregulated the expression of these genes and the protein expression of catalase in the presence of LPS (Figures 5(a) and 5(b)). Nrf2 translocation from the cytoplasm to the nucleus plays a key role in Nrf2 activation and the transcription of antioxidant enzymes. As shown in Figure 5(c), LPS had no effect on the nuclear translocation of Nrf2, while TBE increased Nrf2 protein within the nuclear fraction. In addition, as previous reports suggested that some protein kinases such as PI3K/Akt and AMPK are involved in Nrf2 translocation [16, 19], we examined the effect of TBE on Akt and AMPK pathways. Phosphorylation of Akt and AMPK was slightly increased in LPS-treated cells, but a significant level of Akt and AMPK phosphorylation occurred in cells treated with TBE (Figure 5(d)).

### 3.7. Effects of GA and EA on Antioxidant Enzyme Expression in LPS-Stimulated Macrophages

**Figure 6:**
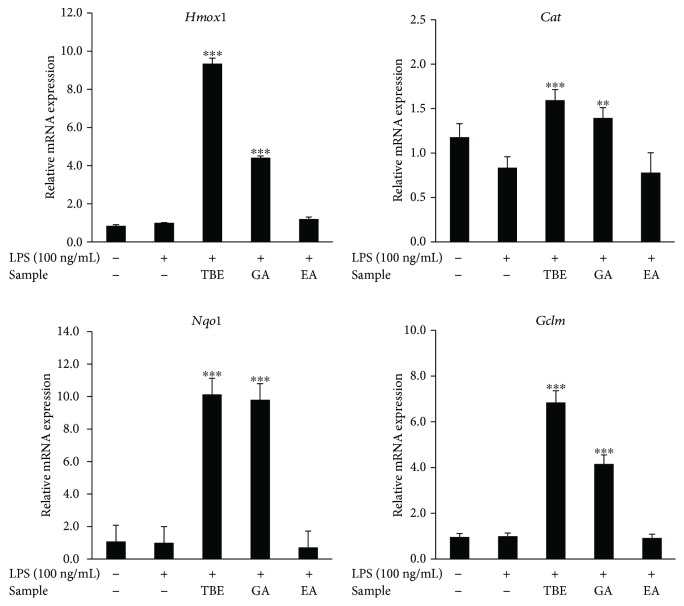
Effects of TBE, GA, and EA on antioxidant enzyme expression in LPS-stimulated macrophages. RAW 264 cells were pretreated with 400 *μ*g/mL TBE, 46 *μ*g/mL GA, or 1.6 *μ*g/mL EA for 1 h, followed by treatment with 100 ng/mL LPS for 4 h. The mRNA levels of *Hmox1*, *Cat*, *Nqo1*, and *Gclm* were detected by real-time RT-PCR. Data represent mean ± SD, *n* = 3 (^∗∗^ *p* < 0.01, ^∗∗∗^ *p* < 0.001 compared to LPS group).

We also analyzed the effects of GA and EA in TBE on antioxidant enzyme expression in LPS-stimulated macrophages. The impacts of TBE, GA, and EA on antioxidant enzyme expression is similar to the effects on inflammatory mediator expression, that is, TBE and GA significantly induced the expression of HO-1, catalase, NQO1, and GCLM, but EA did not affect the expression of these genes (Figure 6).

### 3.8. Blocking Nrf2 Signaling Attenuated the Antioxidant Effects of TBE and GA in LPS-Stimulated Macrophages

**Figure 7:**
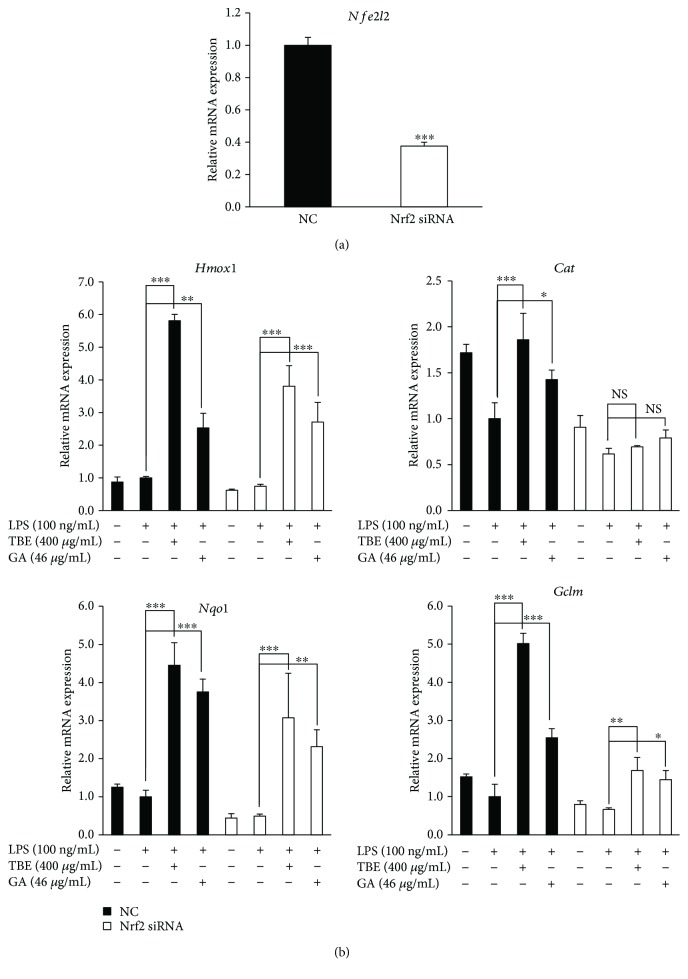
Knockdown of Nrf2 attenuated antioxidant enzyme expression induced by TBE and GA in LPS-stimulated macrophages. (a) RAW 264 cells were transfected with 60 nM Nrf2 siRNA or NC siRNA for 48 h. The mRNA levels of *Nfe2l2* were detected by real-time RT-PCR. Data represent mean ± SD, *n* = 3 (^∗∗∗^ *p* < 0.001 compared to NS group). (b) After transfection of 60 nM Nrf2 siRNA or NC siRNA for 48 h, RAW 264 cells were pretreated with 400 *μ*g/mL TBE or 46 *μ*g/mL GA for 1 h, followed by treatment with 100 ng/mL LPS for 4 h. The mRNA levels of *Hmox1*, *Cat*, *Nqo1*, and *Gclm* were detected by real-time RT-PCR. Data represent mean ± SD, *n* = 3 (^∗^ *p* < 0.05, ^∗∗^ *p* < 0.01, ^∗∗∗^ *p* < 0.001; NS, no significant difference).

Nrf2, a major transcriptional factor regulating the expression of antioxidant enzymes, is involved in the suppression of oxidative stress. To confirm whether the antioxidant effects of TBE and GA are mediated by Nrf2, we silenced Nrf2 gene expression in RAW 264 macrophages. When Nrf2 siRNA was transfected into cells, the level of Nrf2 expression was decreased by approximately 60% compared with cells transfected with NC siRNA (Figure 7(a)). As shown in Figure 7(b), knockdown of Nrf2 significantly inhibited the increase in mRNA expression of catalase and GCLM induced by TBE and GA, without affecting the expression of HO-1 and NQO1. These results show that Nrf2 activation accounts at least in part for the antioxidant effects of TBE and GA in our system.

### 3.9. Involvement of PI3K/Akt and AMPK Pathways in Antioxidant Enzyme Expression by TBE

**Figure 8:**
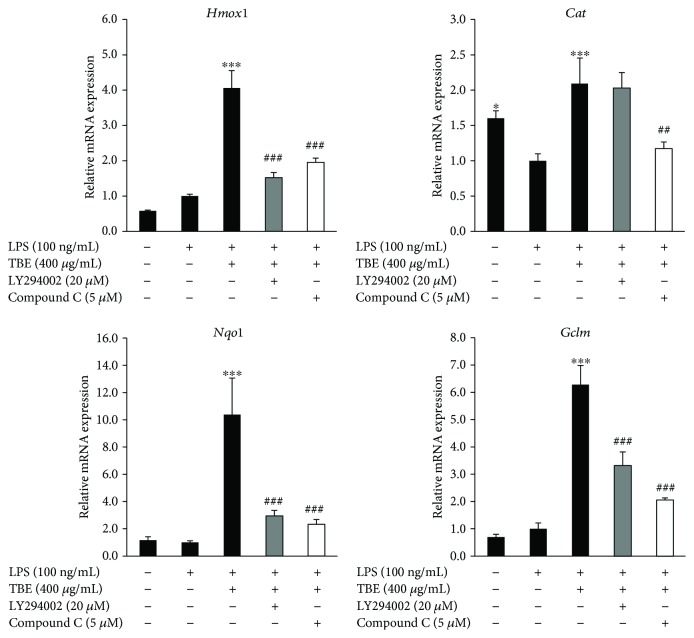
Inhibition of Akt and AMPK reduced TBE-induced antioxidant enzyme expression in LPS-stimulated macrophages. RAW 264 cells were pretreated with 400 *μ*g/mL TBE, 20 *μ*M LY294002, or 5 *μ*M compound C for 1 h, followed by treatment with 100 ng/mL LPS for 4 h. The mRNA levels of *Hmox1*, *Cat*, *Nqo1*, and *Gclm* were detected by real-time RT-PCR. Data represent mean ± SD, *n* = 3 (^∗^ *p* < 0.05, ^∗∗∗^ *p* < 0.001 compared to LPS group; ##*p* < 0.01, ###*p* < 0.001 compared to TBE group).

TBE is capable of activating Akt and AMPK in LPS-stimulated macrophages (Figure 5(d)). To determine whether Akt and AMPK are responsible for the increased expression of antioxidant enzymes induced by TBE, we used LY294002 (Akt inhibitor) and compound C (AMPK inhibitor). As shown in Figure 8, TBE significantly induced the expression of HO-1, catalase, NQO1, and GCLM. However, treatment of cells with LY294002 and compound C resulted in significant inhibition of TBE-induced antioxidant enzyme expression, indicating that the antioxidant effect of TBE is largely dependent on PI3K/Akt and AMPK signaling.

### 3.10. TBE Increased Antioxidant Enzyme Expression and Improved Kidney Injury in LPS-Shock Model Mice

**Figure 9:**
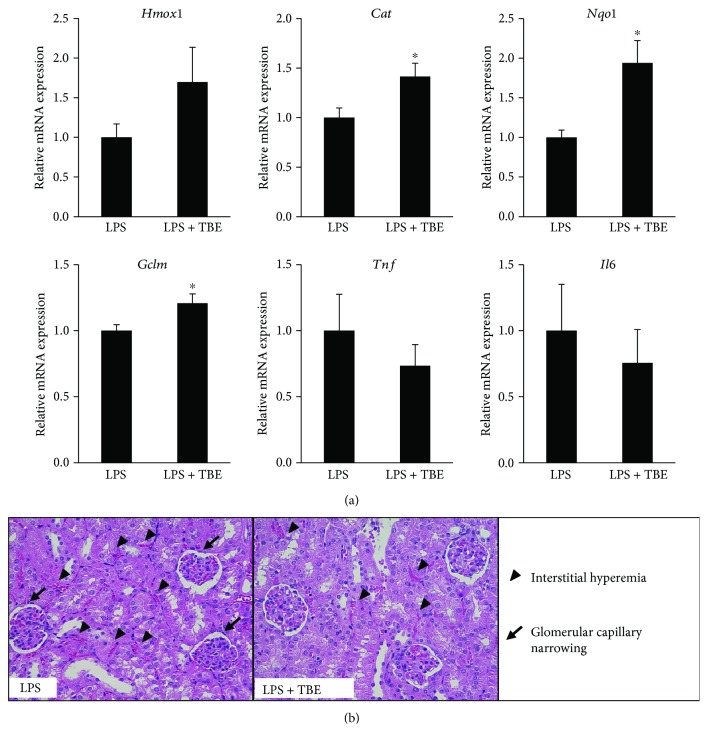
Protective effect of TBE on LPS-induced kidney injury in mice. (a, b) Mice were orally administrated 400 mg/kg TBE or water for 3 days, followed by intraperitoneal injection of 2 mg/kg LPS. Kidney tissues were collected at 24 h post LPS injection. (a) The mRNA levels of *Hmox1*, *Cat*, *Nqo1*, *Gclm*, *Tnf*, and *Il6* were detected by real-time RT-PCR. (b) Histopathological findings of kidney injury were observed by HE staining. Data represent mean ± SE, *n* = 6 (^∗^ *p* < 0.05 compared to LPS group).

As the present data indicate that TBE exhibits antioxidant and anti-inflammatory properties in LPS-stimulated macrophages, we assessed *in vivo* protective effects of TBE in LPS-shock model mice. As shown in Figure 9(a), TBE significantly induced the mRNA expression of antioxidant enzymes (catalase, NQO1, and GCLM) but tended to reduce the mRNA expression of inflammatory mediators (TNF-*α* and IL-6) in kidney tissues. As shown in Figure 9(b), histopathological examination of kidney tissues from the LPS group showed severe lesions including interstitial hyperemia, inflammatory cell infiltration in glomeruli, and glomerular capillary narrowing. In contrast, these kidney injury features were attenuated in the TBE-treated group. The score of interstitial hyperemia was 2.3 ± 0.3 in the LPS group and 1.8 ± 0.1 in the LPS + TBE group. The percentage of glomerular capillary narrowing was 33.2 ± 7.4% in the LPS group and 16.5 ± 7.1% in the LPS + TBE group. These results suggest that TBE might efficiently prevent oxidative stress and inflammation in endotoxemic mice.

## 4. Discussion

Owing to its high polyphenol content, the fruit of *T. bellirica* has been reported to have beneficial effects including antioxidant [33], hypoglycemic [26], and hypolipidemic [27] activities. The present study showed that TBE contains GA and EA and that approximately 50% of the polyphenolic compounds contained in TBE is GA. We demonstrated that TBE and GA attenuated LPS-induced inflammatory mediator overexpression, NO production, ROS production, and NF-*κ*B nuclear translocation in macrophages. Huang et al. [34] reported that GA suppressed LPS-induced inflammatory cytokine production and the mRNA and protein expression of NF-*κ*B, which is partially consistent with our results. We also found that TBE suppressed phosphorylation of not only NF-*κ*B p65 but also p38, JNK, and ERK without altering phosphorylation of I*κ*B. NF-*κ*B p65 is held in its inactive form by association with I*κ*B under quiescent conditions, but phosphorylation and degradation of I*κ*B result in p65 dissociation and nuclear translocation [35]. Although I*κ*B signaling is an established theory of NF-*κ*B activation, recent evidence indicates that phosphorylation of p65 by several kinases is a principal mechanism for NF-*κ*B transcriptional activation. Olson et al. [36] reported that p38 MAPK induced p65 phosphorylation mediated by mitogen- and stress-activated protein kinase 1 (MSK1), as well as subsequent inflammatory cytokine production. Our results indicate that TBE may regulate LPS-induced NF-*κ*B translocation via the suppression of NF-*κ*B p65 phosphorylation mediated by MAPK but not via the suppression of I*κ*B phosphorylation. Additionally, TBE decreased the mRNA and protein expression of SR-A, a major pattern recognition receptor found on macrophages that binds to modified low-density lipoprotein (LDL), as well as PAMPs including LPS [37]. Recent reports have shown that SR-A is required for LPS-induced MAPK and NF-*κ*B activation by cooperating with TLR4 in macrophages [38, 39]. Therefore, we speculated that downregulation of SR-A might contribute to the inhibitory effect of TBE on MAPK/NF-*κ*B inflammatory signaling.

Nrf2 has been reported to regulate cellular redox homeostasis, and antioxidant enzyme production through Nrf2 pathway is one of the major defense mechanisms against oxidative stress and inflammation [14]. Exogenous overexpression of HO-1 and its metabolite carbon monoxide by use of HO-1 inducers inhibited LPS-induced inflammation *in vitro* and *in vivo* [40]. In addition, treatment of catalase inhibitor exacerbated liver injury and led to high lethality from microbial sepsis in mice [41]. In the present study, we found that TBE and GA increased the expression of antioxidant enzymes (HO-1, catalase, NQO1, and GCLM) and activated Nrf2 nuclear translocation in RAW 264 cells. As antioxidant enzymes are produced in response to oxidative stress itself, we assessed intracellular ROS levels. TBE and GA significantly suppressed LPS-induced ROS production, suggesting that antioxidant enzyme expression by TBE and GA might not be mediated by ROS generation. Furthermore, the effects of TBE and GA on catalase and GCLM expression were attenuated when Nrf2 expression was disrupted by siRNA transfection. Several kinases (e.g., PI3K/Akt, AMPK, protein kinase C, and MAPK) are implicated in Nrf2-mediated antioxidant enzyme expression [16, 19]. We also examined the role of PI3K/Akt and AMPK pathways on antioxidant enzyme expression by using specific inhibitors. The present data showed that blockage of PI3K/Akt and AMPK signaling significantly reduced TBE-induced antioxidant enzyme expression, indicating the involvement of PI3K/Akt and AMPK pathway activation in the upregulatory effect of TBE on Nrf2-targeted antioxidant genes. As not only Nrf2 but also other transcription factors such as hypoxia-inducible factor-1*α* [42] and peroxisome proliferator-activated receptors [43] are known to regulate antioxidant gene expression, further research is needed to identify mechanisms underlying antioxidative effect of TBE.

Previous studies have shown that some polyphenols (e.g., resveratrol, quercetin, and catechins) suppressed LPS-induced inflammatory mediator expression via the activation of PI3K/Akt and AMPK [22–24]. However, in the present study, inhibition of PI3K/Akt and AMPK had no effect on decreasing LPS-induced mRNA expression of inflammatory mediators by TBE (data not shown). Thus, the inhibitory effect of TBE and GA on LPS-induced inflammatory response might be mediated by the suppression of MAPK/NF-*κ*B pathway, which occurs independently of PI3K/Akt and AMPK activation.

Finally, we tested the anti-inflammatory and antioxidant properties of TBE in endotoxin-shocked mice. Intraperitoneal injection of LPS has been shown to cause excessive inflammation in whole body organs including the liver, kidney, and lung [44–46]. In the present study, TBE significantly induced antioxidant enzyme expression and improved kidney injury in LPS-shock model mice but had little effect in LPS-induced liver and lung injury (data not shown). Acute kidney injury is one of the most frequent symptoms of sepsis and increases the mortality rate compared with sepsis alone [45]. Recent study suggested that GA was mainly distributed in kidney tissue after oral administration [47], explaining the beneficial effect of TBE to ameliorate kidney injury. GA has also been shown to attenuate dextran sulfate sodium-induced colitis by upregulating Nrf2 pathway and its downstream targets [48]. Therefore, the protective effect of TBE on kidney injury might result from its antioxidant activity.

In conclusion, our present study demonstrated that TBE and GA enhance antioxidant defense capacity through the activation of Akt/AMPK/Nrf2 pathway in LPS-stimulated macrophages. Our data also showed that TBE and GA exhibit anti-inflammatory activities by downregulating MAPK/NF-*κ*B pathway. The *in vivo* efficacy of TBE was partly confirmed in LPS-shock model mice. These findings provide new perspectives for novel therapeutic approaches using dietary-derived antioxidants against oxidative stress and inflammation-related diseases.

## Data Availability

The data are available from the corresponding author upon request.

## Supplementary Materials

Supplementary Materials Table S1: the primer sequences of genes for real-time RT-PCR.
