# Supplementary material for: Terminalia bellirica (Gaertn.) Roxb. Extract and Gallic Acid Attenuate LPS-Induced Inflammation and Oxidative Stress via MAPK/NF-κB and Akt/AMPK/Nrf2 Pathways
Source: Oxid Med Cell Longev. 2018 Nov 8;2018:9364364. doi: 10.1155/2018/9364364 (PMC6250009; doi:10.1155/2018/9364364)
Supplement: Supplementary Materials — Table S1: the primer sequences of genes for real-time RT-PCR. [file 9364364.f1.docx]

**Supplementary Table S1: The primer sequences of genes for real-time RT-PCR**

| **Gene** | **Forward primer (5′ to 3′)** | **Reverse primer (5′ to 3′)** |
| --- | --- | --- |
| *Tnf* | CAAATGGCCTCCCTCTCATC | CTCCAGCTGCTCCTCCACTT |
| *Il1b* | TGAGCACCTTCTTTTCCTTCATC | TGTCTAATGGGAACGTCACACAC |
| *Il6* | ACAACCACGGCCTTCCCTACTT | CACGATTTCCCAGAGAACATGTG |
| *Nos2* | AGGACCACCTCTATCAGGAAGAAA | CAGCTGCTTTTGCAGGATGT |
| *Ccl2* | ATGCTTCTGGGCCTGCTGT | GGATCATCTTGCTGGTGAATGAG |
| *Msr1* | CATGAACGAGAGGATGCTGACT | GGAAGGGATGCTGTCATTGAA |
| *Hmox1* | AAGGGTCAGGTGTCCAGAGAAG | GGGAAGTAGAGTGGGGCATAGA |
| *Cat* | TATTGCCGTTCGATTCTCCAC | CCCACAAGATCCCAGTTACCA |
| *Nqo1* | CTGAAGAAGAGAGGATGGGAGGT | GAGATGACTCGGAAGGATACTGAAA |
| *Gclm* | CCCGATGAAAGAGAAGAAATGAAAG | GTCCAGCTGTGCAACTCCAA |
| *Nfe2l2* | CAGCACATCCAGACAGACACC | AAGCGACTCATGGTCATCTACAAA |
| *Gapdh* | TGACGTGCCGCCTGGAGAAA | AGTGTAGCCCAAGATGCCCTTCAG |
